# Retinal microvasculature features in patients with migraine: a systematic review and meta-analysis

**DOI:** 10.3389/fneur.2023.1187559

**Published:** 2023-09-15

**Authors:** Yulian Pang, Ting Cao, Qinglin Zhang, Haijian Hu, Zhiruo Wang, Jiahe Nie, Ming Jin, Guiping Chen, Xu Zhang

**Affiliations:** ^1^Affiliated Eye Hospital of Nanchang University, Jiangxi Clinical Research Center of Ophthalmic Disease, Jiangxi Provincial Key Laboratory for Ophthalmology, Nanchang, Jiangxi, China; ^2^Department of Orthopaedics, The Fourth Medical Center of Chinese PLA General Hospital, Beijing, China; ^3^Medical School of Chinese PLA, Beijing, China; ^4^Department of Ophthalmology, Huangshi Central Hospital, Edong Healthcare Group, Affiliated Hospital of Hubei Polytechnic University, Huangshi, Hubei, China

**Keywords:** migraine, optical coherence tomography angiography, retinal microvasculature, vascular density, meta-analysis

## Abstract

**Background:**

Migraine is a central nervous system disorder involving neuronal and vascular factors. The brain has a close anatomical relationship with retinal vessels and similar regulatory processes, and the retinal vascular system is the only *in vivo* vessel that can be directly visualized, while optical coherence tomography angiography (OCTA) is an advanced retinal vascular imaging technique. In this study, OCTA was used to study the retinal vascular density (VD) and foveal avascular zone (FAZ) in migraine patients, which provided a theoretical basis for its use as a candidate for rapid and non-invasive diagnosis of migraine.

**Methods:**

Published studies comparing retinal microvascular profiles between migraine patients and healthy controls were obtained by a comprehensive search of electronic databases. Nine studies were finally included, including 775 eyes (migraine group: 444 eyes, control group: 331 eyes). Pooled effect sizes were presented as standardized mean differences (SMDs) and 95% confidence intervals (CIs). Statistical analysis was performed using Review Manager software (version 5.30).

**Results:**

The combined results revealed that the superficial and deep macular whole enface VD (MWEVD) (superficial VD: SMD = −0.30, *P* = 0.0001; deep VD: SMD = −0.61, *P* = 0.02), superficial foveal VD (FVD) (SMD = −0.42, *P* = 0.03), deep parafoveal VD (PFVD) (SMD = −0.31, *P* = 0.002), and peripapillary VD (PVD) (SMD = −0.49, *P* = 0.002) were significantly reduced in migraine patients compared with healthy people. However, there was a significant increase in the area of the FAZ in migraine patients (SMD = 0.56, *P* < 0.0001).

**Conclusion:**

Migraine patients are prone to retinal microcirculation disorders, such as decreased blood vessel density and increased avascular area in the fovea. This provides a theoretical basis for OCTA as a candidate for rapid, non-invasive diagnosis of migraine.

## 1. Introduction

Migraine is a common type of primary headache and an important cause of disability, placing a heavy burden on society and family (1). Its diagnostic criteria refer to the International Classification of Headache (ICHD-3. org/1-migraine) (2). Migraine attacks often go through prodromal, aura, headache, and post-symptomatic periods, with complex and comprehensive symptoms and neurological problems detected at each stage (3). Aura symptoms, which almost always appear before the headache and last for several hours to several days in one-third of migraine sufferers, include visual, sensory, language, or brain stem disorders (4). Cortical spreading depression (CSD) is thought to be the cause of migraine aura (5), characterized by changes in cortical potentials and fluctuations of blood flow (1). CSD leads to sodium, calcium, and water influx, with potassium, protons, glutamate, and ATP flowing from the cells and diffusing interactively between adjacent cells, ultimately activating meningeal nociceptors and the perivascular trigeminal nerve (6). Another view of the trigger pattern of migraine without aura suggests that the hypothalamus, as a possible source of migraine attacks, can be activated by stress, sleep deprivation, and mood changes and transmit these parasympathetic signals to the superior salivary nucleus (SSN), and signals from the SSN activate postganglionic parasympathetic neurons in the sphenopalatine ganglion (SPG) projecting to the meninges to activate meningeal nociceptors and trigeminal vascular pathways (7). The so-called trigeminal vascular pathway refers to the brain with an abundant noxious nerve fiber plexus originating from the trigeminal ganglion and innervating intracranial structures, including the leptomeninges, arachnoid and dural vessels, and cerebral arteries. The axon terminals of these nociceptive nerve fibers contain vasoactive neuropeptides, including calcitonin gene-related peptide, and substance P, which are released after stimulation by signals of CSD or hypothalamic origin, causing vasodilation of the dura mater and leptomeninges, as well as the release of inflammatory cytokines (8). Furthermore, these injury signals are transmitted through afferent nerves in the peripheral trigeminal ganglion, converging on neurons in the trigeminal cervical complex (TCC) after trigeminal ganglion exchange, and ascending connections in the TCC transmit signals to the brainstem, thalamus, hypothalamus, and basal ganglia. Multiple cortical regions process inputs from the TCC, resulting in the development of migraine and phenotypic expression of other associated symptoms (9, 10).

A large body of data suggests an association between migraine and vascular disease. Migraine with aura is an independent risk factor for overall vascular event recurrence and ischemic stroke recurrence in young ischemic stroke (11), and patients with migraine appear to have an increased risk of transient intracerebral hemorrhage (TIA) attacks (12). A recent population-based cohort study showed that migraine aura increases the risk of death from coronary artery disease (13). In addition, some studies have also found that migraine is associated with vascular mortality (14) as well as vascular diseases at other sites (lower limbs, and eyes) (15, 16), such as Raynaud's syndrome, retinal artery occlusion, and glaucoma. Associated mechanisms may rely on vascular susceptibility unique to migraine patients, which may contribute to the pathogenesis of migraine and, over time, may also contribute to the development of other vascular diseases (17).

In embryology, the retina is an extension of the cortex, and their angiogenesis patterns are similar during development (18). Therefore, there is a close anatomical relationship and similar regulatory processes between the blood vessels of the brain and retina (19). With the continued development of retinal vascular imaging techniques, there is increasing interest in using optical coherence tomography angiography (OCTA) to explore the potential retinal involvement of cerebrovascular changes in migraine. OCTA is a technique that uses the movement of red blood cells to detect blood flow in the retinal capillaries and has the advantages of being rapid, non-invasive, and high-resolution in comparison to traditional fundus fluorescence angiography (FFA) (20, 21). Some studies using OCTA have shown increased foveal avascular zone (FAZ) and decreased parafoveal vascular density (PFVD) and peripapillary vascular density (PVD) in migraine patients with aura compared to healthy controls (22, 23). Other studies have found no difference in macular vascular density (VD) in migraine patients with or without aura compared to controls (24). VD was expressed as the area or length of vessels with blood flow in the retina as a percentage of the total measured area. Software will automatically partition and calculate foveal VD (FVD) and PFVD. The division of superficial VD and deep VD varies slightly by software, but in general the superficial retinal capillary network is transversely arranged and forms an interconnected vascular network between the nutrient arterioles and draining venules, which is usually what we can see on conventional FFA (25). The deep capillary network is composed of polygonal units, in which the capillaries radially converge toward the central vortex capillaries and then drain to the superficial venules through the interconnected venules in the vertical direction. The traditional FFA cannot see this layer of the vascular network, but OCTA can show (26). In addition, PVD can assess ischemia and its potential role in glaucoma, and precise details of these capillaries can be seen on OCTA but not on conventional FFA, which is a clear advantage of OCTA over conventional imaging modalities (27). The FAZ refers to the area surrounded by the continuous capillary plexus of the retina without any capillary structure itself, and its morphology and changes in peripheral capillary density reflect the degree of macular ischemia and are commonly used indicators for OCTA to evaluate the severity and progression of angioretinopathy (28). The software automatically calculated the area by clicking on the center of the FAZ. Given these inconsistent results, it is necessary to conduct additional meta-analyses of published studies. Moreover, no meta-analysis has systematically evaluated retinal microvascular characteristics associated with migraine. Therefore, we carried out this study to systematically evaluate the retinal microvascular system in patients with migraine, so as to provide a theoretical basis for OCTA as a candidate for rapid, non-invasive diagnosis of migraine.

## 2. Methods

### 2.1. Databases and search strategy

Reporting Items for Systematic Reviews and Meta-Preferred Analyses (PRISMA) were strictly followed in this study (29). Two researchers (Y-LP and TC) searched PubMed, Embase, the Cochrane Library, and the Web of Science (from inception to 15 January 2023) and selected the research that may be related to this topic. We used “OCTA” and “Migraine” as Medical Subject Headings (MeSH) to develop a search strategy and search for relevant studies. The entire search strategy is shown in Supplementary Table 1. The meta-analysis was registered at PROSPERO (CRD42022338486).

### 2.2. Inclusion and exclusion criteria

The inclusion criteria were as follows: (1) to investigate the characteristics of retinal microvessels in migraine patients; (2) studies using OCTA to detect retinal microvessels; (3) in accordance with the International Classification of Headache (ICHD-3. org/1-migraine) criteria for migraine diagnosis; (4) cross-sectional studies in adults >18 years of age; (5) age-matched healthy people as a control group; (6) OCTA data are provided for superficial and deep panmacular VD (MWEVD), superficial and deep foveal VD (FVD), superficial and deep PFVD, FAZ, and PVD. The exclusion criteria were as follows: (1) any study containing ocular diseases, refractive errors >3.00, and a history of ocular surgery; (2) any study containing neurological disorders other than migraine; (3) any study containing systemic diseases other than migraine and regular medication; (4) duplicate studies retrieved from various databases; (5) study design is a study of case reports, letters, comments, and reviews; (6) studies in animals or children; (7) no studies with extractable data.

### 2.3. Data extraction and transformation

Two authors (YP and TC) extracted data from the selected studies: first author's name, publication year, country, research type, average age, sample size, OCTA brand, main results, and migraine diagnostic criteria. We transformed the data in quartile form into mean ± SD form according to Luo et al. (30) and Wan et al. (31). Data for the migraine with aura and migraine without aura subgroups were combined in reference to the study by Zhang et al. (32) with the following formula: Set the sample size of subgroup A as N1, mean as M1 and standard deviation as SD1; if the sample size of subgroup B is N2, mean as M2 and standard deviation as SD2, the combined sample size N = N1 + N2, M = (N1M1 + N2M2)/(N1 + N2), SD = √[(N_1_-1)SD_1_^2^ + (N_2_-1)SD_2_^2^ + N_1_N_2_/(N_1_ + N_2_) (M_1_^2^ + M_2_^2^−2M_1_M_2_)/(N_1_+N_2_-1)].

### 2.4. Quality evaluation

We referred to the Newcastle–Ottawa scale (NOS) adapted for cross-sectional studies, in order to assess the quality of all the included studies in the meta-analysis (33). It was deemed that studies with a total score of 5 or above were of higher quality (34).

### 2.5. Data analysis

Data analysis was done using Review Manager (RevMan) software (version 5.30) (Cochrane Collaboration, Oxford, UK). Continuous variables were presented as mean ± standard deviation (SDs), and in view of the differences between the OCTA device and the data analysis system used for inclusion in the study, to eliminate the influence of absolute value size and unit of measure on the results, we used a standardized mean difference (SMD) with a 95% confidence interval (CI) to assess the pooled effect size. Sample means and standard deviations were calculated as before (35). Chi^2^ and I^2^ tests were used to assess heterogeneity across studies. A total of 25%, 50%, and 75% of I^2^ values were considered mild, moderate, and high heterogeneity, respectively. Fixed effects models were used if there was no significant heterogeneity between studies; otherwise, random effects models were used. A *P*-value of <0.05 was considered statistically significant in the study.

## 3. Results

### 3.1. Search characteristics

**Figure 1:**
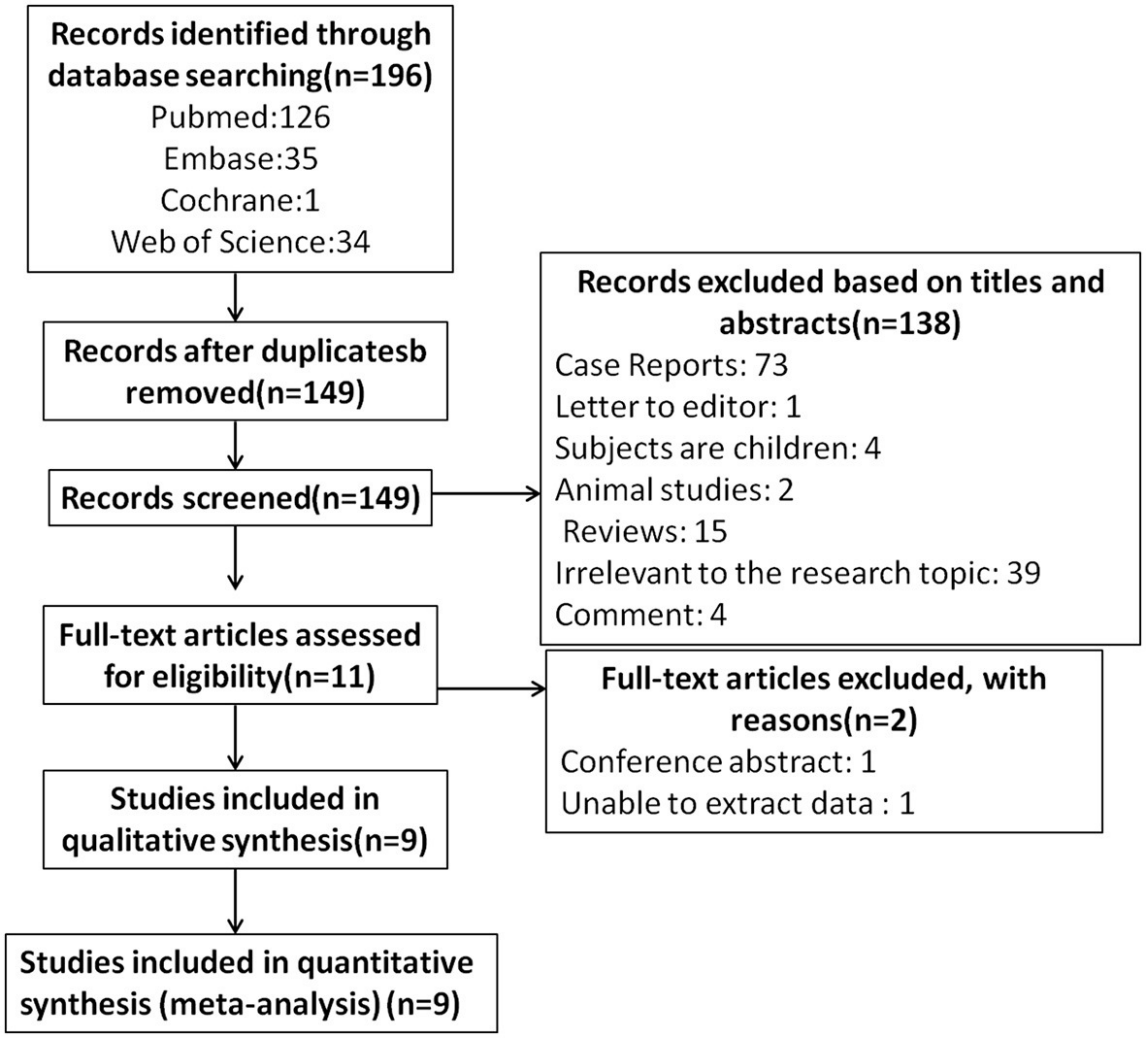
Flow diagram of study selection in the meta-analysis.

The literature screening process is presented in Figure 1. A total of 196 potentially relevant articles were retrieved across all databases. After 47 duplicates were removed, and 138 records were excluded for titles and abstracts. Among the remaining 11 articles, two were further excluded for lack of conference abstract and extractable data. Eventually, nine studies matched the inclusion and exclusion criteria for quantitative meta-analysis (22–24, 36–41), with a total of 775 eyes (migraine group: 444 eyes, control group: 331 eyes). The PRISMA flow diagram is presented in Figure 1. The basic characteristics of the study are shown in Table 1, the quality evaluation is shown in Table 2, and the scores are all above 5.

**Table 1:** Basic characteristics of the included studies.

**References**	**Country**	**Design**	**Age (years)**	**Number**	**OCTA device**	**Scan size (mm^2^)**	**Primary outcomes**	**Diagnostic criteria**
Bingöl et al. (36)	Turkey	Cross-sectional study	MWA:32.1; MWOA:30.4; Control:32.3	MWA:17; MWOA:16; Control:28	Optovue	6 × 6 in macular, 4.5 × 4.5 in ONH	Superficial and deep MWEVD; superficial and deep FVD; superficial and deep PFVD;FAZ; PVD	ICHD
Chang et al. (22)	United States	Cross-sectional study	MWA:42; MWOA:46; Control:39	MWA:15; MWOA:12; Control:22	Optovue	3 × 3 in macular; 3 × 3 in ONH	Superficial and deep MWEVD; superficial and deep FVD; FAZ; PVD	ICHD
Güler et al. (37)	Turkey	Cross-sectional study	MWOA:34.23 ± 11.4; Control:34.63 ± 9.40	MWOA:52; Control:48	Optovue	6 × 6 in macular; 6 × 6 in ONH	Superficial and deep MWEVD; superficial and deep FVD; superficial and deep PFVD	ICHD
Hamamci et al. (23)	Turkey	Cross-sectional study	MWA:33.93 ± 6.42; MWOA:32.23 ± 7.48; Control:34.07 ± 6.52	MWVA:30, MWOVA:30, Control:30	Optovue	6 × 6 in macular, 4.5 × 4.5 in ONH	Superficial and deep MWEVD; superficial and deep FVD; superficial and deep PFVD; FAZ; PVD	ICHD
Hamurcu et al. (38)	Turkey	Cross-sectional study	MWA:37.2 ± 12.8; Control:36.9 ± 11.5	MWA:38; Control:38	Optovue	3 × 3 in macular	FAZ	ICHD
He et al. (39)	China	Cross-sectional study	MWA:40.4 ± 16.2; MWOA:39.3 ± 9.5; Control:38.2 ± 11.6	MWA:23; MWOA:31; Control:32	Zeiss	6 × 6 in macular; 6 × 6 in ONH	Superficial MWEVD; superficial FVD; superficial PFVD; FAZ	ICHD
Karahan et al. (24)	Turkey	Cross-sectional study	MWA:35.16 ± 8.4; Control:33.8 ± 6.7	MWA:60; Controls:56	Optovue	3 × 3 in macular; 4.5 × 4.5 in ONH	Superficial and deep MWEVD; superficial and deep FVD; superficial and deep PFVD; FAZ; PVD	ICHD
Tasli et al. (40)	Turkey	Cross-sectional study	MWOA:38.00 ± 7.66; Control:36.88 ± 8.23	MWOA:66, Control:43	Nidek	3 × 3 in macular; 2.4 × 4 in ONH	Superficial and deep MWEVD; FAZ	ICHD
Ulusoy et al. (41)	Turkey	Cross-sectional study	MWA:40.8 ± 12.5; MWOA:38.6 ± 13.5 Control:37.1 ± 11.1	MWA:28, MWOA:26, Control:34	Optovue	6 × 6 in macular; 6 × 6 in ONH	Superficial and deep MWEVD; superficial and deep FVD; superficial and deep PFVD; FAZ; PVD	ICHD

MWA, migraine with aura; MWOA, migraine without aura; ONH, optic nerve head; VD, vessel density; MWEVD, macular whole enface VD; FVD, foveal VD; PFVD, parafoveal VD; PVD, peripapillary VD; FAZ, Foveal avascular zone; ICHD, International Classification of Headache Disorders.

**Table 2:** NOS quality assessment for the included studies.

**Methodological item for non-randomized studies (No. 1–8)**	**Bingöl et al. (36)**	**Chang et al. (22)**	**Güler et al. (37)**	**Hamamci et al. (23)**	**Hamurcu et al. (38)**	**He et al. (39)**	**Karahan et al. (24)**	**Tasli et al. (40)**	**Ulusoy et al. (41)**
1. Is the case definition adequate?	1	1	1	1	1	1	1	1	1
2. Representativeness of the cases	1	1	1	1	1	1	1	1	1
3. Selection of controls	0	0	0	0	0	0	0	0	0
4. Definition of controls	1	1	1	1	1	1	1	1	1
5. Comparability of cases and controls on the basis of the design or analysis	2	2	2	2	2	2	2	2	2
6. Ascertainment of exposure	0	0	0	0	0	0	0	1	0
7. Same method of ascertainment for cases and controls	1	1	1	1	1	1	1	1	1
8. Non-response rate	0	0	0	0	0	0	0	0	0
Total score	6	6	6	6	6	6	6	7	6

NOS, Newcastle–Ottawa scale. The selection area included Nos. 1–4, which was up to one score in one question. The comparability area included No. 5, which was up to 2 scores in the question. The exposure area included Nos. 6–8, which was up to one score in one question. The total score was 9.

### 3.2. The superficial and deep MWEVD between migraine and controls

**Figure 2:**
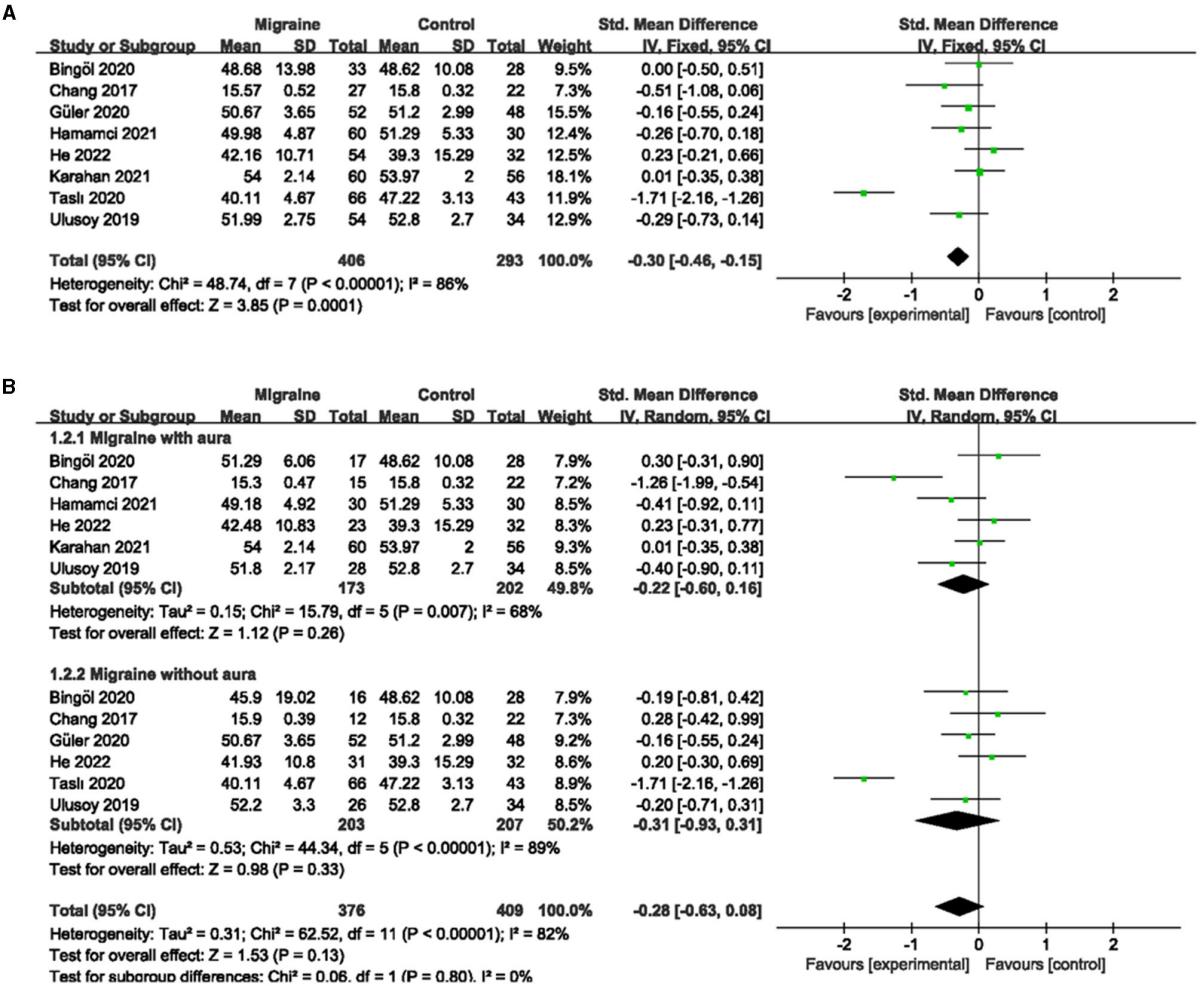
Forest plot for the superficial MWEVD. **(A)** Forest plot for the superficial MWEVD between migraine and control groups. **(B)** Forest plot for the superficial MWEVD between migraine with/without aura and the control groups.

**Figure 3:**
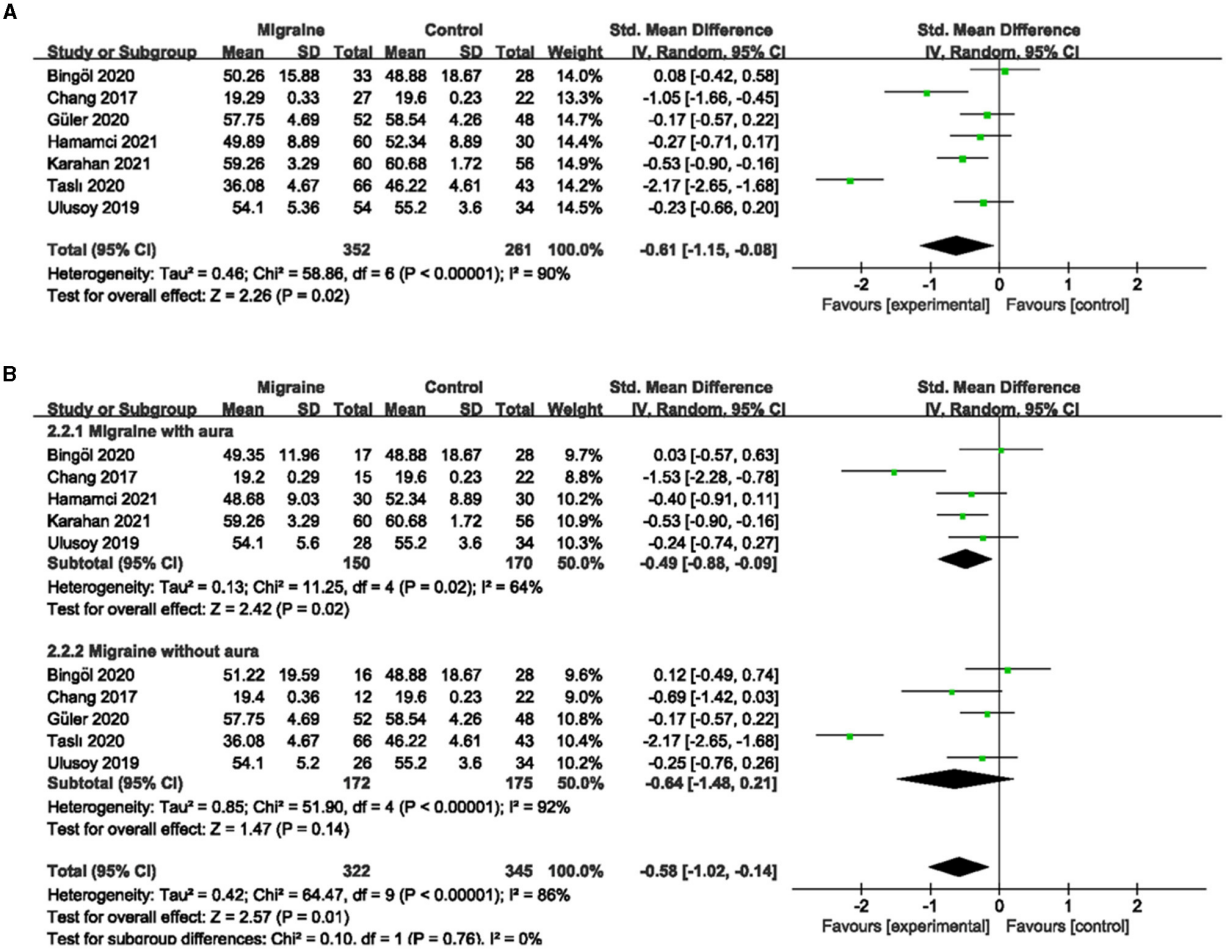
Forest plot for the deep MWEVD. **(A)** Forest plot for the deep MWEVD between migraine and control groups. **(B)** Forest plot for the deep MWEVD between migraine with/without aura and control groups.

A total of 8 studies, including 699 eyes (migraine group: 406 eyes, control group: 293 eyes), reported superficial MWEVD. Meta-analysis showed that the combined SMD was −0.30 (95% CI: −0.46 to −0.15, *P* = 0.0001; Figure 2A), indicating that migraine patients had significantly lower superficial MWEVD than controls, and the included studies had high heterogeneity (Chi^2^ = 48.74, *P* < 0.00001, *I*^2^ = 86%; Figure 2A). The deep MWEVD was reported in 7 studies, including 613 eyes (migraine group: 352 eyes, control group: 261 eyes). Meta-analysis showed that the combined SMD was −0.61 (95% CI: −1.15 to −0.08, *P* = 0.02; Figure 3A), showing that migraine also had significantly lower deep MWEVD than controls. The heterogeneity of the included studies was moderate (Chi^2^ = 58.86, *P* < 0.00001, *I*^2^ = 90%; Figure 3A). According to the analysis, the higher heterogeneity came from the study by Taşlí et al. (40). and may be due to the fact that the OCTA equipment (Nidek) used in this study was different from several other studies. However, due to the small number of included studies, we could not perform subgroup analyses for different instruments.

Subgroup analyses were performed for superficial and deep MWEVD across migraine subtypes. Six studies, including 375 eyes (migraine group: 173 eyes, control group: 202 eyes), reported superficial MWEVD in migraine with aura and controls. The pooled SMD was −0.22 (95% CI: −0.60 to 0.16, *P* = 0.26; Figure 2B), showing no significant change in the superficial MWEVD between the two groups. The heterogeneity of the included studies was moderate (Chi^2^ = 15.79, *P* = 0.007, *I*^2^ = 68%; Figure 2B). Five studies, including 320 eyes (migraine group: 150 eyes, control group: 170 eyes), reported deep MWEVD in migraine with aura and controls. The combined SMD in both groups was −0.49 (95% CI: −0.88 to −0.09, *P* = 0.02; Figure 3B), which showed a significant reduction in the deep MWEVD in migraine with the aura group compared to the control group, which also showed moderate heterogeneity in the included studies (Chi^2^ = 11.25, *P* = 0.02, *I*^2^ = 64%; Figure 3B). Six studies, including 410 eyes (migraine group: 203 eyes, control group: 207 eyes), reported superficial MWEVD in migraine without aura and controls. The combined SMD was −0.31 (95% CI: −0.93 to 0.31, *P* = 0.33; Figure 2B), showing no significant change in the superficial MWEVD between the two groups. The included studies were highly heterogeneous (Chi^2^ = 44.34, *P* < 0.00001, *I*^2^ = 89%; Figure 2B). Five studies, including 347 eyes (migraine group: 172 eyes, control group: 175 eyes), reported deep MWEVD in migraine without aura and controls. The combined SMD in both groups was −0.64 (95% CI: −1.48 to 0.21, *P* = 0.14; Figure 3B), showing no significant change in the deep MWEVD between the two groups. The included studies were highly heterogeneous (Chi^2^ = 51.90, *P* < 0.00001, *I*^2^ = 92%; Figure 3B).

### 3.3. The superficial and deep FVD between migraine and controls

**Figure 4:**
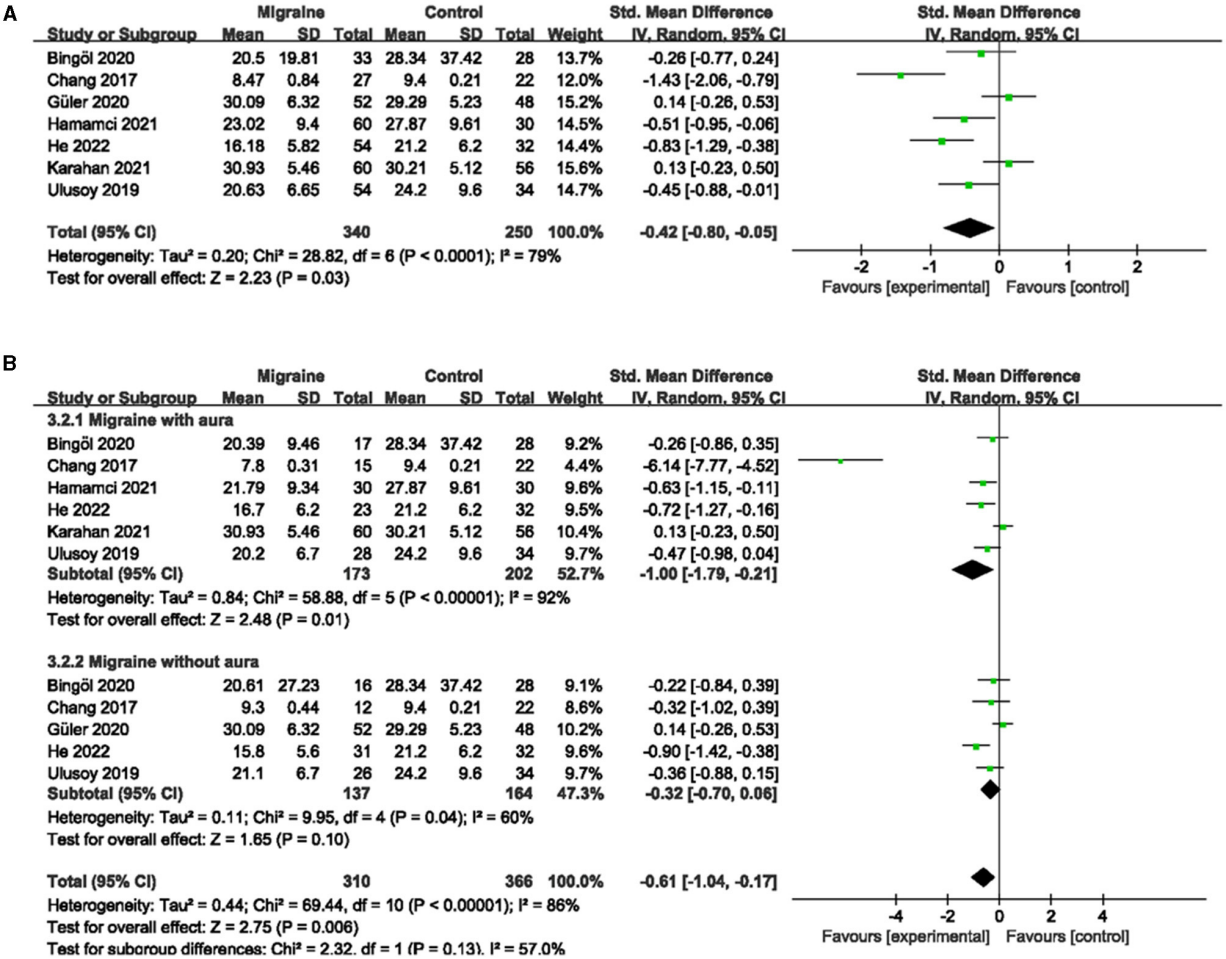
Forest plot for the superficial FVD. **(A)** Forest plot for the superficial FVD between migraine and control groups. **(B)** Forest plot for the superficial FVD between migraine with/without aura and control groups.

**Figure 5:**
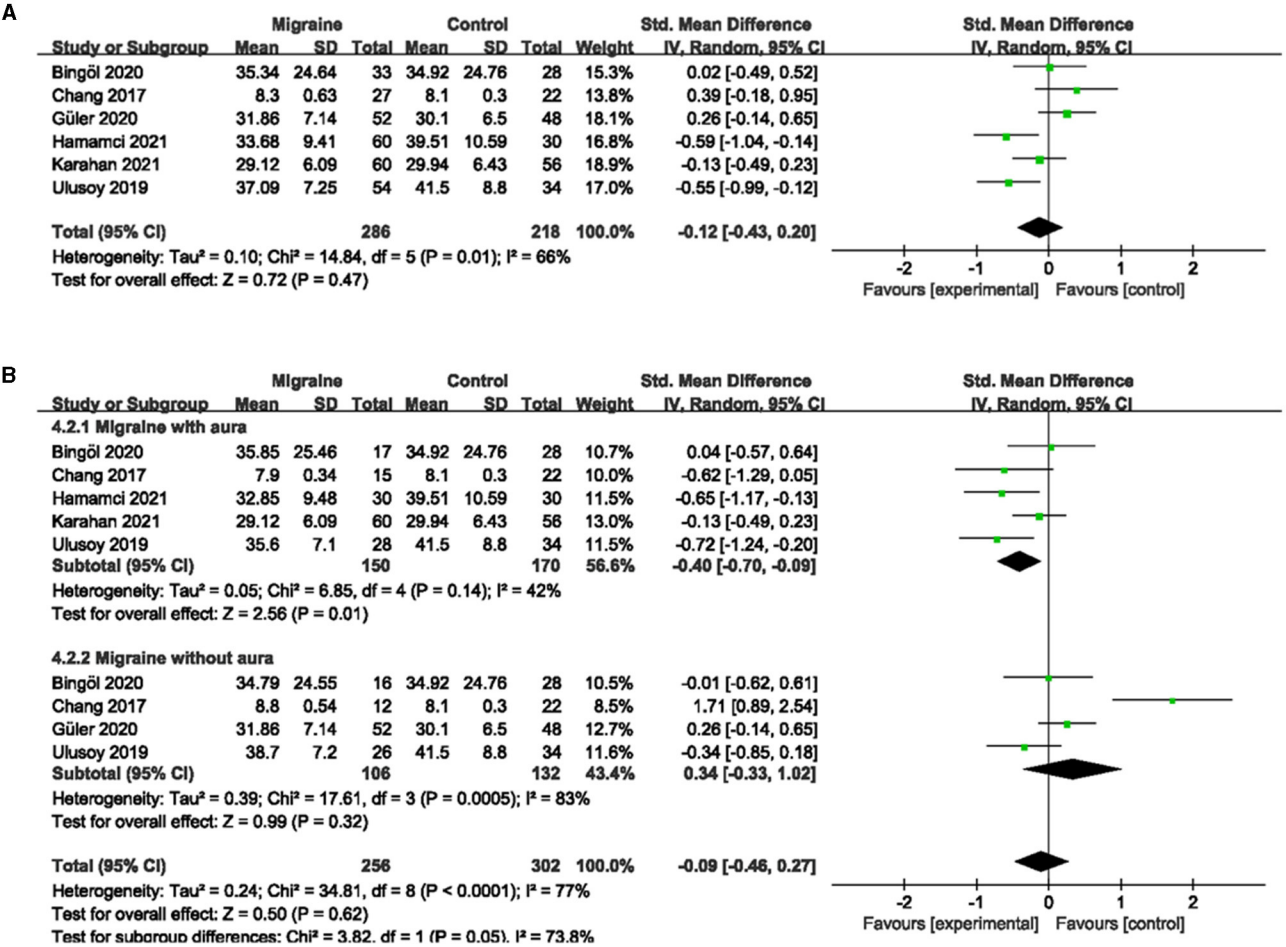
Forest plot for the deep FVD. **(A)** Forest plot for the deep FVD between migraine and control groups. **(B)** Forest plot for the deep FVD between migraine with/without aura and control groups.

A total of 7 studies, including 590 eyes (migraine group: 340 eyes, control group: 250 eyes), reported superficial FVD. Meta-analysis showed that the combined SMD of the migraine and control groups was −0.42 (95% CI: −0.80 to −0.05, *P* = 0.03; Figure 4A), indicating that migraine patients had significantly lower superficial FVD than controls, and the included studies had high heterogeneity (Chi^2^ = 28.82, *P* < 0.0001, *I*^2^ = 79%; Figure 4A). The deep FVD was reported in 6 studies, including 504 eyes (migraine group: 286 eyes, control group: 218 eyes). Meta-analysis showed that the combined SMD of migraine and control groups was −0.12 (95% CI: −0.43 to −0.20, *P* = 0.47; Figure 5A), showing no significant change in the deep FVD between the two groups. The heterogeneity of the included studies was moderate (Chi^2^ = 14.84, P = 0.01, *I*^2^ = 66%; Figure 5A).

Subgroup analyses were performed for the superficial and deep FVD across migraine subtypes. Six studies, including 375 eyes (migraine group: 173 eyes, control group: 202 eyes), reported superficial FVD in migraine with aura and controls. The combined SMD in both groups was −1.00 (95% CI: −1.79 to −0.21, P = 0.01; Figure 4B) showed a significant reduction in the superficial FVD in the migraine with aura group compared to the control group. The included studies were highly heterogeneous (Chi^2^ = 58.88, *P* < 0.00001, *I*^2^ = 92%; Figure 4B). Five studies, including 320 eyes (migraine group: 150 eyes, control group: 170 eyes), reported deep FVD in migraine with aura and controls. The combined SMD in both groups was −0.40 (95% CI: −0.70 to −0.09, *P* = 0.01; Figure 5B), which showed a significant reduction in the deep FVD in migraine with aura group compared to the control group, which also showed moderate heterogeneity in the included studies (Chi^2^ = 8.85, *P* = 0.14, *I*^2^ = 42%; Figure 5B). Five studies, including 301 eyes (migraine group: 137 eyes, control group: 164 eyes), reported superficial FVD in migraine without aura and controls. The combined SMD was −0.32 (95% CI: −0.70 to 0.06, *P* = 0.10; Figure 4B), showing no significant change in the superficial FVD between the two groups. The heterogeneity of the included studies was moderate (Chi^2^ = 9.95, *P* = 0.04, *I*^2^ = 60%; Figure 4B). Four studies, including 238 eyes (migraine group: 106 eyes, control group: 132 eyes), reported deep FVD in migraine without aura and controls. The combined SMD was 0.34 (95% CI: −0.33 to 1.02, *P* = 0.32; Figure 5B), showing no significant change in the deep FVD between the two groups. The included studies were highly heterogeneous (Chi^2^ = 17.61, *P* = 0.0005, *I*^2^ = 83%; Figure 5B).

### 3.4. The superficial and deep PFVD between migraine and controls

**Figure 6:**
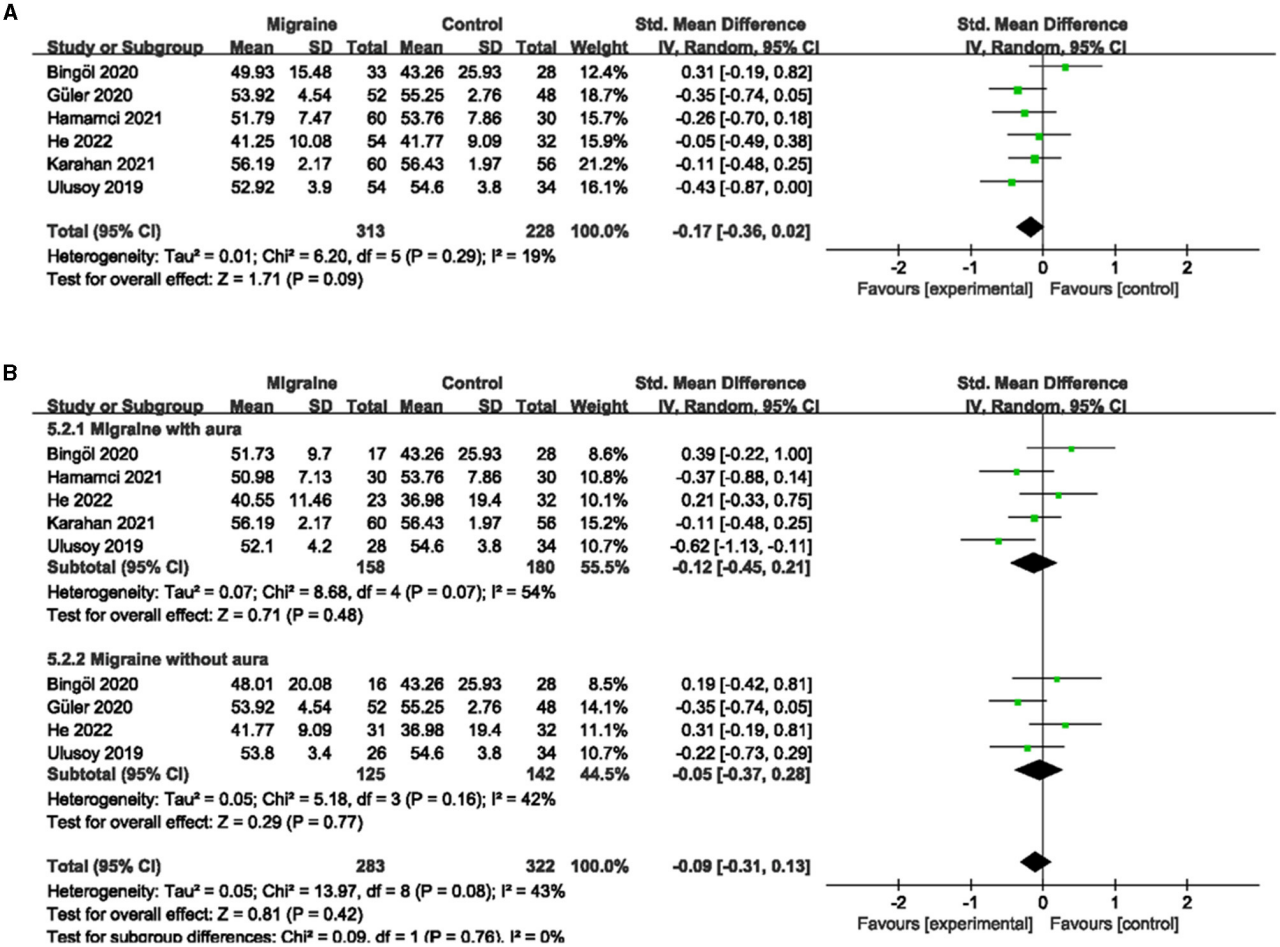
Forest plot for the superficial PFVD. **(A)** Forest plot for the superficial PFVD between migraine and control groups. **(B)** Forest plot for the superficial PFVD between migraine with/without aura and control groups.

**Figure 7:**
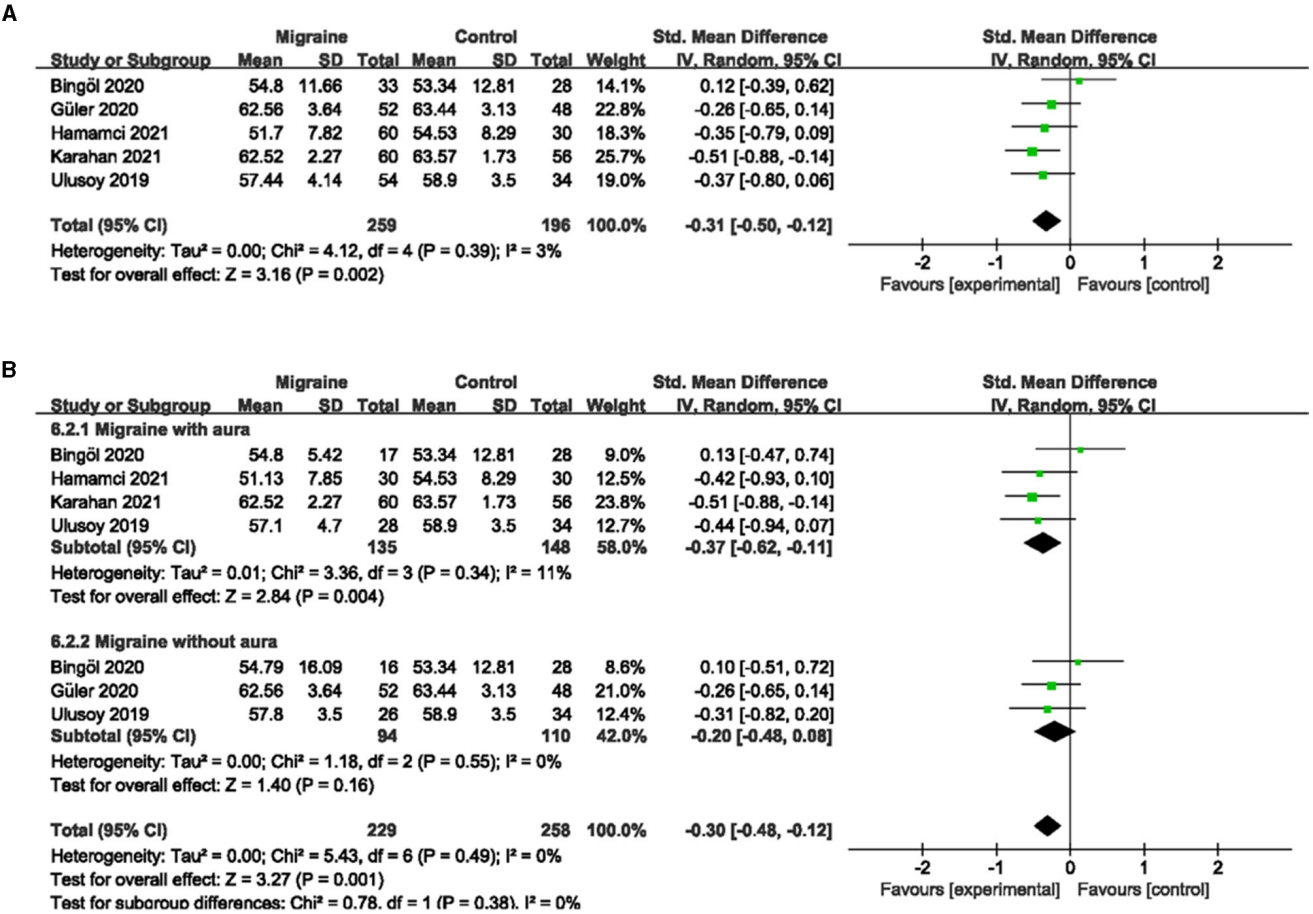
Forest plot for the deep PFVD. **(A)** Forest plot for the deep PFVD between migraine and control groups. **(B)** Forest plot for the deep PFVD between migraine with/without aura and control groups.

A total of six studies, including 541 eyes (migraine group: 313 eyes, control group: 228 eyes), reported superficial PFVD. Meta-analysis showed that the combined SMD of the migraine and control groups was −0.17 (95% CI: −0.36 to 0.02, *P* = 0.09; Figure 6A), showing no significant change in the superficial PFVD between the two groups, and the included studies had low heterogeneity (Chi^2^ = 6.20, *P* =0.29, *I*^2^ = 19%; Figure 6A). Deep PFVD was reported in five studies, including 455 eyes (migraine group: 259 eyes, control group: 196 eyes). Meta-analysis showed that the combined SMD of migraine and control groups was −0.31 (95% CI: −0.50 to −0.12, *P* = 0.002; Figure 7A), indicating that migraine patients had significantly lower deep PFVD than controls, with low heterogeneity in the included studies (Chi^2^ = 4.12, *P* = 0.39, *I*^2^ = 3%; Figure 7A).

Subgroup analyses were performed for the superficial and deep PFVD across migraine subtypes. Five studies, including 338 eyes (migraine group: 158 eyes, control group: 180 eyes), reported the superficial PFVD in migraine with aura and controls. The combined SMD was −0.12 (95% CI: −0.45 to 0.21, *P* = 0.48; Figure 6B), showing no significant change in the superficial PFVD between the two groups. The included studies were moderately heterogeneous (Chi^2^ = 8.68, *P* = 0.07, *I*^2^ = 54%; Figure 6B). Four studies, including 283 eyes (migraine group: 135 eyes, control group: 148 eyes), reported deep PFVD in migraine with aura and controls. The combined SMD was −0.37 (95% CI: −0.62 to −0.11, *P* = 0.004; Figure 7B), which showed a significant reduction in deep PFVD in migraine with aura group compared to the control group, which also presented low heterogeneity in the included studies (Chi^2^ = 3.36, *P* = 0.34, *I*^2^ = 11%; Figure 7B). Four studies, including 267 eyes (migraine group: 125 eyes, control group: 142 eyes), reported the superficial PFVD in migraine without aura and controls. The combined SMD was −0.05 (95% CI: −0.37 to 0.28, *P* = 0.77; Figure 6B) showing no significant change in the superficial PFVD between the two groups. The heterogeneity of the included studies was moderate (Chi^2^ = 5.18, *P* = 0.16, *I*^2^ = 42%; Figure 6B). Three studies, including 204 eyes (migraine group: 94 eyes, control group: 110 eyes), reported deep PFVD in migraine without aura and controls. The combined SMD was −0.20 (95% CI: −0.48 to 0.08, *P* = 0.16; Figure 7B) showing no significant change in the deep PFVD between the two groups. Studies included were not heterogeneous (Chi^2^ = 1.18, *P* =0.55, *I*^2^ = 0%; Figure 7B).

### 3.5. The PVD between migraine and controls

**Figure 8:**
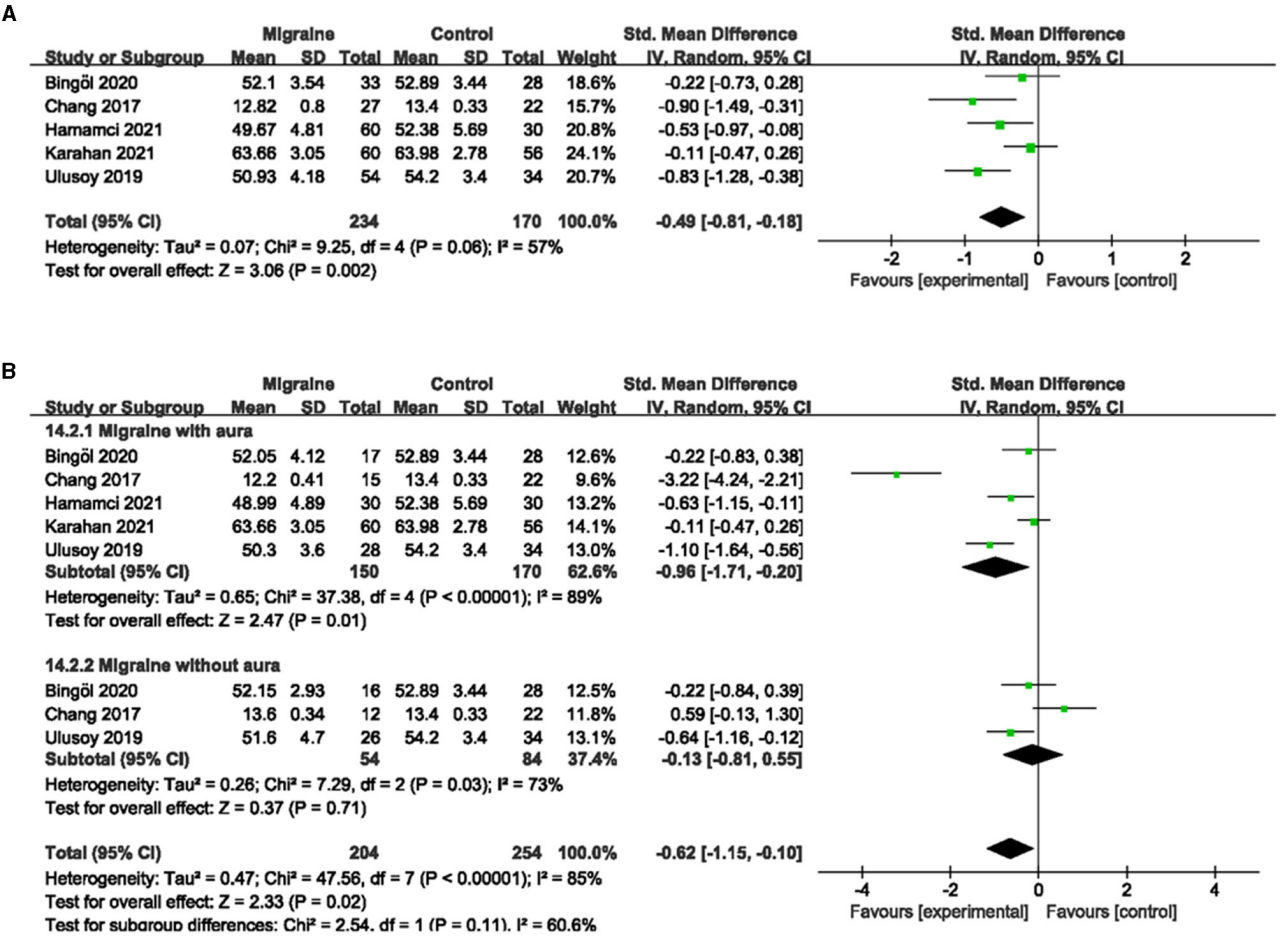
Forest plot for the PVD. **(A)** Forest plot for the PVD between migraine and control groups. **(B)** Forest plot for the PVD between migraine with/without aura and control groups.

Five studies, including 404 eyes (migraine group: 234 eyes, control group: 170 eyes), reported the PVD. Combined SMD in migraine and controls was −0.49 (95% CI: −0.81 to −0.18, *P* = 0.002; Figure 8A), showing significantly lower PVD in migraine patients than in controls. The heterogeneity of the included studies was moderate (Chi^2^ = 9.25, *P* = 0.06, I^2^ = 57%; Figure 8A).

Subgroup analyses were performed for the PVD across migraine subtypes. Five studies, including 320 eyes (migraine group: 150 eyes, control group: 170 eyes), reported PVD in migraine with aura and controls. The combined SMD was −0.96 (95% CI: −1.71 to −0.20, *P* = 0.01; Figure 8B), showing a significant reduction in PVD in migraine with aura group compared to the control group. The heterogeneity of the included studies was high (Chi^2^ = 37.38, *P* < 0.00001, *I*^2^ = 89%; Figure 8B). Three studies, including 138 eyes (migraine group: 54 eyes, control group: 84 eyes), reported PVD in migraine without aura and controls. The combined SMD was −0.13 (95% CI: −0.81 to 0.55, *P* = 0.71; Figure 8B), showing no significant change in the PVD between the two groups. The heterogeneity of the included studies was high (Chi^2^ = 7.29, *P* = 0.03, *I*^2^ = 73%; Figure 8B).

### 3.6. The FAZ analysis between migraine and controls

**Figure 9:**
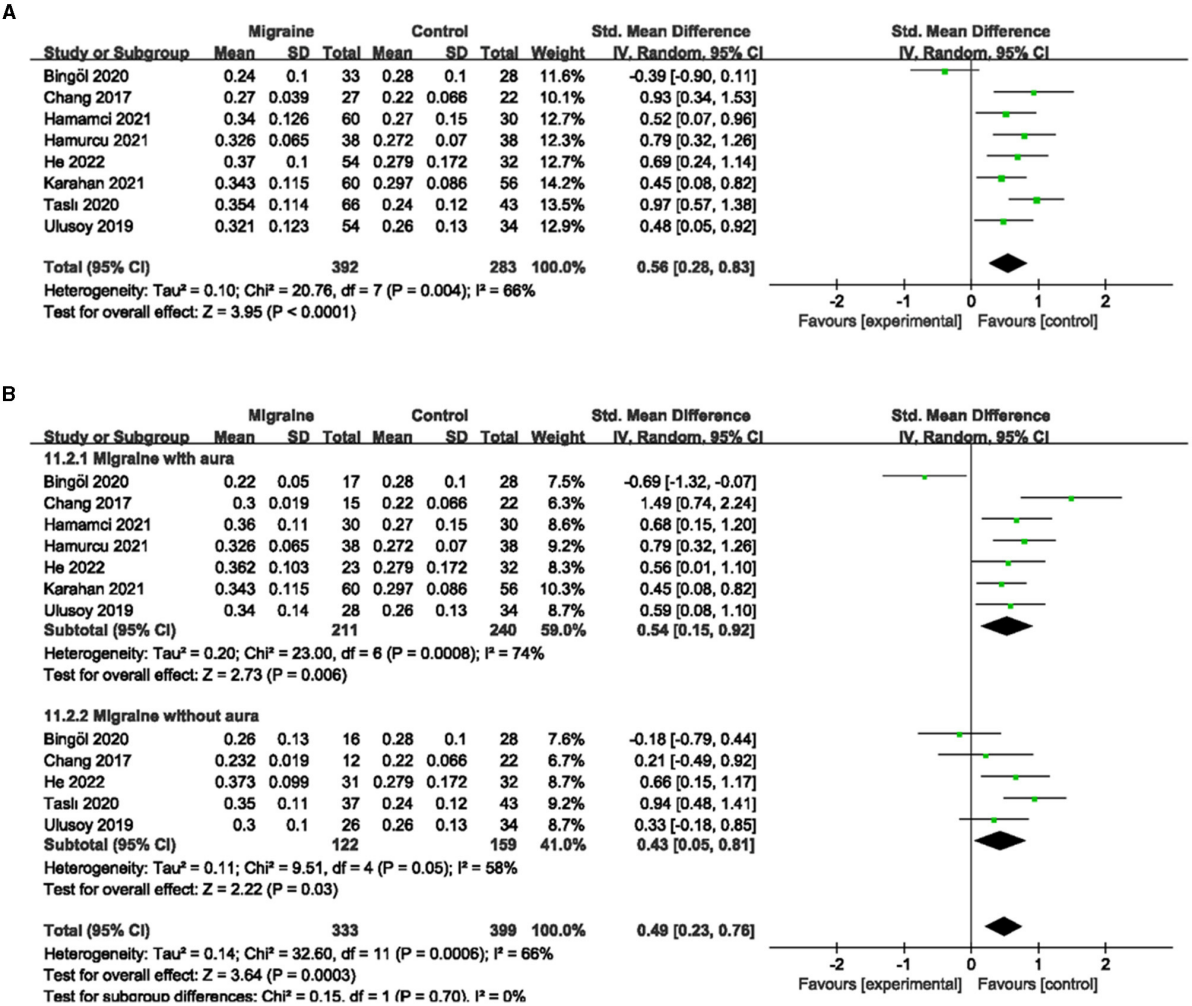
Forest plot for FAZ. **(A)** Forest plot for FAZ between migraine and control groups. **(B)** Forest plot for FAZ between migraine with/without aura and control groups.

A total of eight studies reported FAZ area, of which studies by Hamurcu et al. and Ulusoy et al. did not address the specific site of FAZ measurement (superficial or deep), and studies by Chang et al. and Hamamci et al. used a single combined quantitative measurement based on the PR-OCTA algorithm. The studies by Karahan et al., Bingöl et al., He et al., and Taslí et al. used shallow and deep measurements. Given the limited number of studies overall, if meta-analysis according to the measurement method classification was of little significance, we finally decided to include eight studies, including 675 eyes (migraine group: 392 eyes, control group: 283 eyes) for the analysis of combined FAZ. The results showed that the combined SMD was 0.56 (95% CI: 0.28 to 0.83, *P* < 0.0001; Figure 9A), indicating that the FAZ area was significantly larger in migraine patients than in controls. The heterogeneity of the included studies was moderate (Chi^2^ = 20.76, *P* = 0.004, *I*^2^ = 66%; Figure 9A).

Subgroup analyses were performed for FAZ across migraine subtypes. Seven studies, including 451 eyes (migraine group: 211 eyes, control group: 240 eyes), reported FAZ area in migraine with aura and control groups, with a combined SMD of 0.54 (95% CI: 0.15 to 0.92, *P* = 0.006; Figure 9B), indicating a significantly larger FAZ area in migraine with aura than in controls. The heterogeneity of the included studies was moderate (Chi^2^ = 23.00, *P* = 0.0008, *I*^2^ = 74%; Figure 9B). Five studies, including 281 eyes (migraine group: 122 eyes, control group: 159 eyes), reported FAZ area in migraine without aura and controls, with a combined SMD of 0.43 (95% CI: 0.05 to 0.81, *P* = 0.03; Figure 9B) showing a significantly larger FAZ area in migraine without aura than in controls. The heterogeneity of the included studies was moderate (Chi^2^ = 9.51, *P* = 0.05, *I*^2^ = 58%; Figure 9B).

## 4. Discussion

Migraine is a complex, multifactorial neurological disorder whose main clinical symptom is unilateral pulsatile headache with photosensitivity and sound sensitivity (42). Migraine patients often seek help from ophthalmologists in the presence of visual aura and periocular pain, which has aroused interest and concern among researchers who try to explore the underlying mechanisms of this disease using technical means in the ophthalmology field to provide new insights into the early diagnosis and treatment of the disease (43, 44). OCTA is an exciting new imaging modality that uses motion contrast to provide non-invasive images of retinal and choroidal vascular structures and can provide detailed features beyond conventional imaging (45). Therefore, in this meta-analysis, we chose to include nine available studies that used OCTA to investigate retinal microvascular characteristics in migraine patients (migraine group: 444 eyes, control group: 331 eyes). The results of our analysis showed that patients with migraine presented significantly decreased superficial and deep MWEVD, superficial FVD, deep PFVD, and PVD, while the FAZ area was significantly increased compared to healthy controls. In addition, migraine patients with aura had significantly lower deep MWEVD, superficial and deep FVD, and deep PFVD than healthy controls, whereas migraine patients without aura did not differ significantly. These results lead us to further understand retinal microangiopathy in migraine patients and whether aura or not may be closely related to the degree of lesions, which will provide new thinking and opportunities for the observation and research of migraine.

Migraine is a nervous system disease involving neuronal and vascular mechanisms (46). Although the exact mechanism remains uncertain, the trigeminovascular system is undeniably critical, and evidence supports CSD as a potential physiological process contributing to trigeminal activation in migraine (47). CSD is a large depolarizing wave propagating in gray matter, which leads to synaptic activity, changes in extracellular ion concentrations, and the release of calcitonin gene-related peptide (CGRP) from trigeminal nerve endings (48). CGRP as the main driver of neurogenic meningeal vasodilation in migraine eventually leads to an increase in regional cerebral blood flow (49), which is called spreading congestion and lasts approximately 1–2 min. This is followed by prolonged hypoperfusion lasting 1–2 h, which is called spreading ischemia (50). These changes in cerebral blood flow and oxygenation, as well as the increased metabolic demands associated with CSD, lead to a supply and demand mismatch, further aggravating the development of ischemia and subsequent parenchymal lesions (51). In migraineurs, alterations in gray matter in several cortical regions related to pain circuits have been observed, which may be central nervous system remodeling as a result of repeated chronic pain and ischemia (52, 53). Moreover, migraineurs are more prone to white matter hyperintensities (WMHs) than healthy controls, implying that migraineurs may suffer microvascular damage, leading to the disruption of axonal integrity (54). In addition, a large body of data suggests that there is systemic impairment in the vasculature of migraine patients, and migraine patients experience changes in arterial function, such as stiffness or impaired compliance of the arterial system, relative to non-migraine patients (55). It has been found that patients with interictal migraine have abnormal blood flow patterns similar to Raynaud's phenomenon (56), and increased retinal microcirculation resistance in studies (57, 58). Repeated retinal vasospasm can lead to permanent ocular sequelae, such as retinal artery occlusion and visual field defects (59, 60). Given the embryohistological association of the brain and retina mentioned in the introduction, it is possible to better manage migraine if microvascular changes in migraine can be monitored non-invasively early by OCTA.

Migraine is a risk factor for retinal and optic ischemic disease (61). Greven et al. reported a history of migraine in 25% of youth with retinal artery occlusion (62). Kara's et al. color Doppler ultrasonography examination also indicated a significant increase in the resistance of retinal arteries in migraine patients (63). In this study, the results of a meta-analysis of retinal macular VD showed that the superficial and deep MWEVD, superficial FVD, and deep PFVD were significantly reduced. Our results strongly support the theory of retinal microangiopathy in migraine patients. Long-term recurrent retinal vasospasm stenosis in migraine patients eventually leads to a decrease in vascular density. We also noticed that the deep FVD also showed a trend to decrease compared to healthy people but there was no significant statistical difference. This may be due in part to projection artifacts, which introduce errors in the assessment of VD in the deep retina (64), while the limited number of included studies resulting in a small sample size may be another important reason. In addition, studies have shown that migraine patients with aura more often experience ischemic events than migraine patients without aura (65). CSD (electrophysiological basis of aura) has been shown to increase the risk of cerebral ischemia in mice carrying a mutated human migraine gene (66). Therefore, we further performed a subgroup analysis of retinal VD with and without aura. The results showed that migraine patients with aura had significantly lower deep MWEVD, shallow and deep FVD, and deep PFVD than healthy controls. However, there was no statistically significant difference in retinal VD in migraine patients without aura although there was also a decreasing trend. This result strongly supports the clear association of migraine aura with ischemic events. Migraine relies on the activation and sensitization of the trigeminovascular pain pathway, whereas cortical spreading depression (CSD) is thought to be associated with the neurophysiology of migraine aura (55). CSD may induce injury by hypoperfusion (diffuse hypoperfusion), activation of the inflammatory cascade, and failure of neurovascular coupling to provide adequate blood flow (5). Most existing studies have shown that migraine with aura can be considered a risk factor for ischemic stroke, whereas migraine without aura is not considered (17). In addition, data from the Women's Health Study (WHS) suggest that patients with migraine aura have an increased risk of transient ischemic attack (TIA), compared with patients without migraine aura (67). Our results further suggest that there may be systemic impairment of the vascular system in migraine with aura. This finding emphasizes that the diagnosis and treatment of migraine should not only focus on pain, but monitoring the occurrence of aura symptoms may become a key link in preventing and judging the prognosis of the disease. In addition, our study also found a significant increase in the FAZ area in migraineurs. Migraine as a recurrent chronic disease can cause permanent structural abnormalities in the brain and even the retina, and changes in the FAZ region may be caused by vascular remodeling caused by long-term recurrent retinal capillary ischemia (17, 68, 69). Our findings provide further evidence for the theory of retinal microangiopathy in migraine patients.

In addition to studying the changes in the VD in the macular area, we also analyzed the VD in the optic disk area. Our results indicated that the PVD was significantly lower in migraineurs. Several earlier studies suggested that the PVD reduction in migraine patients is significant for optic nerve diseases (anterior and posterior ischemic optic neuropathy, etc.) (70). Migraine is also associated with normal-pressure glaucoma, and many epidemiological and case–control studies have investigated the potential link between glaucoma and migraine (71, 72). Their results indicated a close association between migraine and glaucoma (73–75). In addition, the PVD has been shown to be reduced in patients with normal-tension glaucoma, and the degree of reduction correlates with disease severity (76). Therefore, it is reasonable to think that the PVD reduction mediates the association between migraine and normal-tension glaucoma, which is consistent with RNFL thinning in structural OCT studies of migraine and glaucoma (77). Chronic peripapillary hypoperfusion eventually leads to RNFL atrophy and thinning.

The development of modern ophthalmic imaging techniques provides an opportunity to observe the characteristics of retinal microvessels in migraine patients and also makes it possible to observe systemic vascular changes in migraine through such a visual window in the retina. However, most existing reports are cross-sectional studies. Thus, the results do not indicate whether the observed retinal microvascular abnormalities are indicative of systemic vasculopathy as a cause of migraine or a result of migraine recurrence, whereas the small number of relevant cross-sectional studies led to the limited sample size included in this analysis. Future studies should better specify the type (location and severity) of retinal microangiopathy in migraine patients, identify the subgroups of migraine corresponding to different types and their association with prognosis, and identify the time span from migraine onset to retinal microvascular abnormalities. In addition, it is necessary to further explain the mechanism of the association between migraine and retinal microvascular abnormalities. Specifically regarding the role of genetic predisposing factors or co-morbidity triggers, this is a starting point to try to develop any reliable strategy to monitor and prevent possible risks. At the same time, in view of the relatively new nature of OCTA, the lack of reliable systematic analysis methods across imaging platforms also has an impact on the accuracy of summary analysis between different devices. Therefore, it is necessary to standardize the measurement and analysis methods of OCTA in future.

## 5. Conclusion

Overall, our study provides strong evidence for retinal microangiopathy in migraine patients and further finds a close link between retinal microangiopathy and migraine aura. Future studies should further clarify the specific association of OCTA findings with ocular and systemic vascular events in migraine to make OCTA a powerful tool for rapid, non-invasive monitoring of migraine and its aura symptoms.

## Author contributions

YP and TC conceived and designed the study. YP, TC, and XZ searched the manuscript. YP and QZ contributed to data acquisition and analysis. HH, ZW, and JN were responsible for the software. YP and TC contributed to the writing of the original manuscript. MJ, GC, and XZ were responsible for revising and reviewing. All authors read and approved the final manuscript.
