# Supplementary material for: Retinal microvasculature features in patients with migraine: a systematic review and meta-analysis
Source: Front Neurol. 2023 Sep 15;14:1187559. doi: 10.3389/fneur.2023.1187559 (PMC10540451; doi:10.3389/fneur.2023.1187559)
Supplement: Supplementary file 1 [file Table_1.docx]

| Electronic databases searched | PubMed (http://www.ncbi.nlm.nih.gov/PubMed/), Embase (http://www.embase.com), Cochrane Library (http://www.thecochranelibrary.com/), Web of Science (http://webofknowledge.com/WOS) |
| --- | --- |
| Search terms for Pubmed was conducted to identify qualified literature from inception to January 15, 2023 | (OCTA OR ‘optical coherence tomography angiography’ OR ‘OCT angiography’ OR ‘optical coherence tomographic angiography’) AND (‘Migraine Disorders’ OR ‘Disorder, Migraine’ OR ‘Disorders, Migraine’ OR ‘Migraine Disorder’ OR ‘Migraine’ OR ‘Migraines’ OR ‘Migraine Headache’ OR ‘Headache, Migraine’ OR ‘Headaches, Migraine’ OR ‘Migraine Headaches’ OR ‘Acute Confusional Migraine’ OR ‘Acute Confusional Migraines’ OR ‘Migraine, Acute Confusional’ OR ‘Migraines, Acute Confusional’ OR ‘Status Migrainosus’ OR ‘Hemicrania Migraine’ OR ‘Hemicrania Migraines’ OR ‘Migraine, Hemicrania’ OR ‘Migraines, Hemicrania’ OR ‘Migraine Variant’ OR ‘Migraine Variants’ OR ‘Variant, Migraine’ OR ‘Variants, Migraine’ OR ‘Sick Headache’ OR ‘Headache, Sick’ OR ‘Headaches, Sick’ OR ‘Sick Headaches’ OR ‘Abdominal Migraine’ OR ‘Abdominal Migraines’ OR ‘Migraine, Abdominal’ OR ‘Migraines, Abdominal’ OR ‘Cervical Migraine Syndrome’ OR ‘Cervical Migraine Syndromes’ OR ‘Migraine Syndrome, Cervical’ OR ‘Migraine Syndromes, Cervical’) |
| Other sources | The references of included articles were searched and reviewed to recognize any relevant literatures. |
